# Exosomal miRNA-mediated intercellular communications and immunomodulatory effects in tumor microenvironments

**DOI:** 10.1186/s12929-023-00964-w

**Published:** 2023-08-21

**Authors:** Howida M. Nail, Chien-Chih Chiu, Chung-Hang Leung, Mahmoud M. M. Ahmed, Hui-Min David Wang

**Affiliations:** 1 Graduate Institute of Biomedical Engineering, National Chung Hsing University, No. 145, Xingda Rd., South Dist., Taichung City, 402 Taiwan; 2 Department of Biotechnology, Kaohsiung Medical University, Kaohsiung, 807 Taiwan; 3 State Key Laboratory of Quality Research in Chinese Medicine, Institute of Chinese Medical Sciences, University of Macau, Taipa, 999078 Macao China; 4 Department of Soil and Environmental Sciences, National Chung Hsing University, 404 Taichung City, Taiwan; 5 Graduate Institute of Medicine, College of Medicine, Kaohsiung Medical University, Kaohsiung, 807 Taiwan; 6 Department of Medical Laboratory Science and Biotechnology, China Medical University, Taichung City, 404 Taiwan

**Keywords:** Exosomes, Biogenesis, miRNA, Immune cells, Cancer, Tumor microenvironment

## Abstract

Extracellular communication, in other words, crosstalk between cells, has a pivotal role in the survival of an organism. This communication occurs by different methods, one of which is extracellular vesicles. Exosomes, which are small lipid extracellular vesicles, have recently been discovered to have a role in signal transduction between cells inside the body. These vesicles contain important bioactive molecules including lipids, proteins, DNA, mRNA, and noncoding RNAs such as microRNAs (miRNAs). Exosomes are secreted by all cells including immune cells (macrophages, lymphocytes, granulocytes, dendritic cells, mast cells) and tumor cells. The tumor microenvironment (TME) represents a complex network that supports the growth of tumor cells. This microenvironment encompasses tumor cells themselves, the extracellular matrix, fibroblasts, endothelial cells, blood vessels, immune cells, and non-cellular components such as exosomes and cytokines. This review aims to provide insights into the latest discoveries concerning how the immune system communicates internally and with other cell types, with a specific focus on research involving exosomal miRNAs in macrophages, dendritic cells, B lymphocytes, and T lymphocytes. Additionally, we will explore the role of exosomal miRNA in the TME and the immunomodulatory effect.

## Background

### Exosomes

Extracellular vesicles (EVs) are divided into two basic groups: ectosomes and exosomes. Ectosomes are vesicles formed by the straight outward budding of the plasma membrane, which results in microvesicles, microparticles, and giant vesicles with diameters ranging from 50 to 1 mm. Exosomes, on the other hand, are endosomal in origin and range in diameter from 30 to 150 nm (on average 100 nm). A particular feature of exosomes in biology is their creation, which may determine their composition, as well as possibly their function, once secreted into the extracellular environment [1]. Exosomes are involved in the biology of many diseases. By transferring and presenting exosomes, exosomes can modulate immune responses and inflammation of antigen peptides, to induce the expression of inflammatory genes in recipient cells [2]. Several metabolic diseases are associated with exosomes, including metabolic disorders related to adipocytes and islet cells [3, 4]. As well as inhibiting neurotoxic oligomers, exosomes can also contribute to neurodegeneration. The development of tumors, angiogenesis, metastasis, sensitivity to chemotherapy, and immune evasion are also associated with exosomes [5, 6].

### Biogenesis of exosomes

**Fig. 1:**
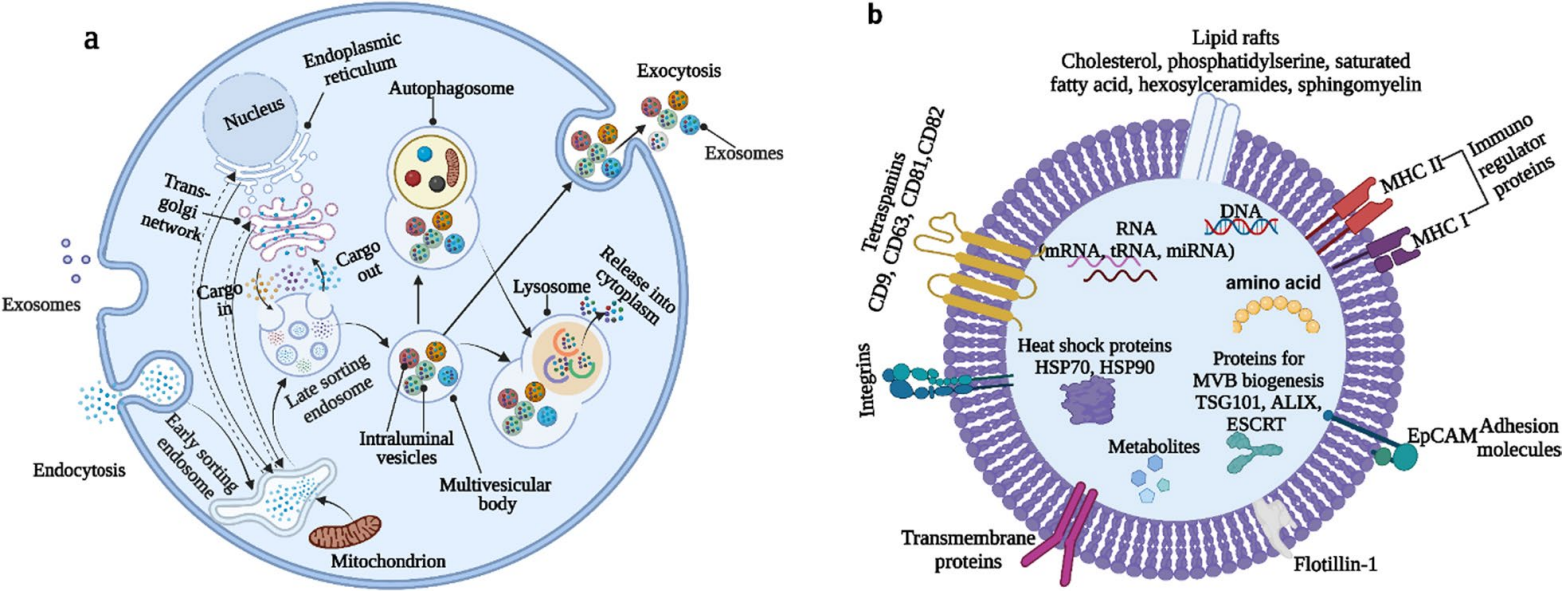
Biogenesis of Exosomes: Exosomes are formed through the invagination of the plasma membrane in two phases. **a** The first phase involves budding formation, which incorporates cell surface proteins, extracellular soluble proteins, and other small molecules, resulting in the formation of early sorting endosomes (ESE). ESEs then mature and convert into late-sorting endosomes (LSE). The second phase involves inward budding of the endosomal limiting membrane, which incorporates constituents from the endoplasmic reticulum (ER), trans-Golgi network (TGN), as well as small biomolecules such as nucleic acids and mitochondria. This process leads to the formation of multivesicular bodies (MVBs). Subsequently, intracellular vesicles (exosomes) are gradually released into the microenvironment after MVBs fuse with the plasma membrane. Additionally, MVBs can fuse with lysosomes or autophagosomes for the degradation of their contents, which can be recycled by the cells. **b** The structure of exosomes: Exosomes contain a wide range of molecules, including nucleic acids, proteins, metabolites, and lipids. Both the membrane-bound components and the soluble components of exosomes represent the cellular origin from which they were released

Intracellular multivesicular bodies (MVBs) containing intraluminal vesicles (ILVs) are formed after two invaginations of the plasma membrane to form exosomes. These ILVs are finally released as exosomes ranging from 30 to 150 nm in diameter by MVB combination to the plasma membrane and exocytosis. During the initial invagination of the plasma membrane, a semi-circular structure is formed that contains proteins associated with the extracellular environment and cell surface proteins. The result of this is the formation of early-sorting endosomes (ESEs) from scratch or sometimes may combine with an already existing ESE. Moreover, the trans-Golgi network, the endoplasmic reticulum plays a significant role in the formation of the ESE. Multivesicular endosomes (MVBs) are produced when ESEs mature into late-sorting endosomes (LSEs). MVBs are formed by a second invagination of the plasma membrane. This is caused by the invagination of the endosomal limiting membrane inward. This process results in the formation of MVBs containing multiple ILVs (future exosomes). The MVBs either combine with lysosomes or autophagosomes to destroy or with the plasma membrane to release the contents of ILVs as exosomes [7–10]. This biogenesis of exosomes discriminates exosomes from EVs that develop via outward budding of the plasma membrane, apoptotic bodies, or necrotic blebs of the plasma membrane. Immune cells, mesenchymal stem cells, fibroblasts, neurons, endothelial cells (ECs), and epithelium cells all secrete exosomes (Fig. 1a).

## The structure of exosomes

After secretion of exosomes from the cell surface, the exosomes have the ability to transport their contents to the cytoplasm of recipient cells by fusing with the plasma membrane. This process, called intracellular signaling, occurs by engaging the proteins on the surface of exosomes with cell surface protein on the recipient cell [11]. Therefore, exosome contents play a crucial role in determining their effect. Firstly, the exosomes plasma membrane is a phospholipid bilayer, the lipid consists of cholesterol, phosphatidylserine, saturated fatty acids, hexosylceramides, and sphingomyelin [11]. The proteome of exosomes includes endosomal, nuclear proteins, cytosolic, and plasma [12]. Exosome-enriched proteins include those involved in membrane delivering and fusing, such as Rab GTPases and annexins, as well as those involved in exosome biogenesis (ESCRT complex, ALIX, TSG101). Moreover, exosomes enriched with integrins, MHC class II proteins, tetraspanins (CD9, CD63, CD81, and CD82), epithelial cell adhesion molecules (EpCAM), heat shock proteins (HSP70, HSP90), and members of the human epidermal receptor (HER) family [13–15]. The protein constituents of exosomes reflect the biogenesis pathway and cell origin. Also, exosomes contain nucleic acids such as many types of RNAs including mRNA, transfer RNAs (tRNAs), viral RNA, and noncoding RNAs such as microRNA (miRNAs) and lncRNAs which are functional and can influence the transcription process in the recipient cells [16]. Exosomes contain small fragments of single-stranded DNA [17] and large fragments of double-stranded DNA [18] (Fig. 1b).

### Communication between cells via exosomes

Intercellular communication occurs due to the presence of connections such as gap junctions or through the process of cell signaling. Signaling occurs when chemical mediators or bioactive molecules are released by one cell and affect other nearby cells. For example, hormones and enzymes released by one cell affect another at a distance [19]. There is also a distinctive way for cells to communicate with distant cells via extracellular vesicles. These membrane vesicles have the ability to transfer lipids, proteins, and genetic materials and function as cargo [20]. Mathieu et al. describe exosomes as small lipid bilayer vesicles carrying different molecules called cargo, which includes proteins, glycans, lipids, metabolites, mRNA, RNA, and DNA. When specific cells secreted exosomes that can act on receptor cells at distal sites and transfer their cargo. These display many of the features of classical endocrine signaling, therefore changing receptor cell phenotypes. Communication between cells has a pivotal role in the cell’s coordination and functioning effectively. This communication between cells can be initiated by exosomes because they have the capability of signal transduction [21].

There are several mechanisms involved in the uptake of exosomes by recipient cells, including endocytosis, micropinocytosis, and phagocytosis. The mechanism of micropinocytosis involves membrane invaginations that pinch off and draw extracellular contents into the cytosol (e.g., fluid and exosomes). The process of endocytosis can be mediated by clathrins or caveolins, and cholesterol-rich microdomains in the cell membrane may facilitate this. The phagocytosis process of exosomes mostly depends on PI3k, which is carried out most efficiently by macrophages. The last way of exosome uptake is by binding directly to the recipient cell membrane and emptying its contents. These mechanisms to transfer information between cells emphasize the different functions of exosomes including immunosuppression, antigen presentation, tumor cell proliferation, autophagy, and apoptosis [22]. As most body cells secret exosomes, therefore it can be found in a wide variety of bodily fluids and can affect neighboring cells functionally. According to some studies, miRNAs make up to 50% of the total noncoding RNA cargo within EVs, depending on the cellular origin of the EV [23–25]. The extracellular miRNAs transfer needs to occur via exosomes as it prevents degradation by free-floating RNases. In this review, we will focus on the questions surrounding the exosomal miRNA to understand their role in the crosstalk between the immune cells and the immunomodulatory effects of exosomal miRNA inside the tumor microenvironments.

## miRNA of exosomes

Exosomes, as previously described, include a vast range of molecules, including proteins, lipids, DNAs, and RNA. Noncoding RNAs, which include miRNAs, tRNA fragments, and Y-RNAs, are the largest RNA component in exosomes, and long noncoding RNAs [26]. Of these molecules, miRNA is the most bioactive molecule that attracts much interest because of its regulatory roles in gene expression. Goldie et al. discovered that the percentage of miRNA in exosomes is greater than in their parent cells [27]. miRNAs are a type of tiny, noncoding RNA with a length of 17–24 nt that intermediate post-transcriptional gene silencing by 3ʹ-binding to the untranslated region (UTR) or open reading frame (ORF) region of target mRNA [28]. Also, many biological processes involving miRNAs have been thoroughly characterized, including cell proliferation, differentiation, migration, disease initiation, and progression. miRNAs are stable in bodily fluids such as saliva, urine, breast milk, and blood. Extracellular miRNAs can be loaded into high-density lipoprotein (HDL) or bound by AGO2 protein outside of vesicles besides being packed into exosomes or microvesicles that prevent miRNA from degradation and ensure their stability [29].

### Biogenesis of miRNA

Inside the nucleus, miRNA biogenesis starts when miRNA of DNA is transcribed by RNA polymerase II to primary miRNA (pri-miRNA). These pri-miRNAs undergo the transcription process as fragments of larger molecules, which are then processed in the nucleus into hairpin RNAs of 70–100 nt by, the double-stranded RNA-specific ribonuclease, Drosha. Hairpin pre-miRNAs are processed by Dicer, a double-stranded-specific ribonuclease, after being moved to the cytoplasm by exportin 5. Double-stranded miRNAs are transformed into single-stranded miRNAs after maturation, then sorted into exosomes in various ways.

There are four potential routes for miRNA sorting into exosomes that have been discovered but these mechanisms are not fully understood. The neural sphingomyelinase 2-dependent (nSMase2) pathway is the first, which enhances the production of exosomal miRNA [30]. The heterogeneous nuclear ribonucleoprotein (hnRNP) dependent pathway is the second, which regulates exosomal miRNA entrance. The miRISC-associated pathway is the third, in this pathway, the mature miRNAs are incorporated into an RNA-induced silencing complex (RISC) along with argonaute (AGO2) and glycine-tryptophan protein of 182 kDa (GW182). AGO2 primarily attaches to U or A at the end of miRNA, which is critical in facilitating the formation of the miRNA: mRNA complex and then destroying or inhibiting translation of the mRNA molecule [31]. The sequence-dependent pathway is the fourth one, which involves the 3ʹ-end of miRNA [32]. The previous studies showed that the distribution of miRNAs in the B cells and their secreted exosomes are not random. miRNAs with an adenylated 3`-end are more abundant in cells, but those with a uridylated 3`-end are more abundant in exosomes. This suggests that the 3`-end of miRNA post-transcriptional modification has an important role in directing miRNA into the exosome [33].

## Immune cells communicate via exosomal miRNAs

### Dendritic cells

Dendritic cells (DCs) are specialized antigen-presenting cells that play critical roles in the initiation and modulation of the innate and adaptive immune systems. DCs are leukocytes generated from bone marrow (BM) and are the most powerful form of antigen-presenting cells. Therefore, when pathogens or pathogen-associated molecular patterns (PAMPs), such as lipopolysaccharide (LPS) invade our body, the mature DCs have the ability to stimulate the adaptive immune system, including T cells. This stimulation occurs through communications between DCs and neighboring cells in different ways such as cytokine signaling, intracellular contact, and vesicle exchange [34]. DC vesicle studies are a novel field because immature and mature bone marrow-derived DCs (BMDCs) are able to exert a huge number of exosomes, which is decreased upon maturation. This trait gives a foundation for understanding the basic cell biology of how exosomes are formed, transported, and internalized [35].

Extracellular miRNA transport via exosomes and the release of exosomes containing different miRNAs by DCs is dependent on their maturation level. Extracellular miRNAs are able to transfer into many different cell types. Because previous studies showed that dye-labeled BMDC exosomes may be taken up by splenic B cells, macrophages, plasmacytoid DCs, and conventional (CD11c-high) DCs in vivo, but not by T cells, both CD4 + and CD8 + subsets. On the other hand, the internalization of immature DC-derived labeled vesicles by CD4 + T cells was achievable in vitro, showing the disparities between in vivo and in vitro experiments within the field, as well as the influence of vesicle-producing cell maturation on the receiving cell. When B cells take up exosomes of mature DC, causes the priming of naïve T cells [36]. EVs of DCs contain different types of noncoding RNAs, which change in response to immune-stimulatory or immune-suppressive. These changes include Y-RNAs, small nucleolar RNAs (snoRNAs), and miRNAs which increased inside DCs in response to any stimulus. This demonstrates how the parent cell state may be reflected in the exosomes generated. This is not always the case, as evidenced by studies that examined large populations of RNA species detected in exosomes [37]. In the field of molecular biology, exosome-mediated miRNA transfer has gained special attention as a means of regulating response between cells, including immune cells. miRNAs, for example, may be shuttled between dendritic cells (DCs) and cause target knockdown in recipient cells, in addition, to conveying at the immunological synapse between T cells and DCs. Further, mice receiving exosomal miRNA have resistance to hepatitis B and aided IFN-α antiviral responses.

Previous studies have shown that DCs communicate with each other through exosomal miR-155, miR-146a, miR-148a, and miR-451. Exosomal miRNAs taken up by receipt cells cause suppression of target genes and can modify the cellular response to endotoxin, for example, miR-155 upregulates while miR-146a downregulates the expression of inflammatory genes [38]. Furthermore, activated bystander T cells can stimulate monocyte-derived DCs to produce vesicle miR-155, resulting in further T cell activation [39]. Alexander M. et. al. found that exosomes of DC contain miR-146a, an anti-inflammatory miRNA, and miR-155, a pro-inflammatory miRNA, taken up by receipt DCs in vitro resulting in modifying their target genes SHIP1, BACH1, Traf6, and IRAK-1 [38]. Hematopoietic cells can also transfer specific miRNAs between themselves, changing their responses. Monocytes can be differentiated into immature DCs and secrete IL-12P70 after receiving mature-derived EVs. These EVs-derived immature DCs play a role in the skewing of T cells, especially for Th1 rather than Th2, this capability is lost after the maturation of EVs-derived DCs.

**Fig. 2:**
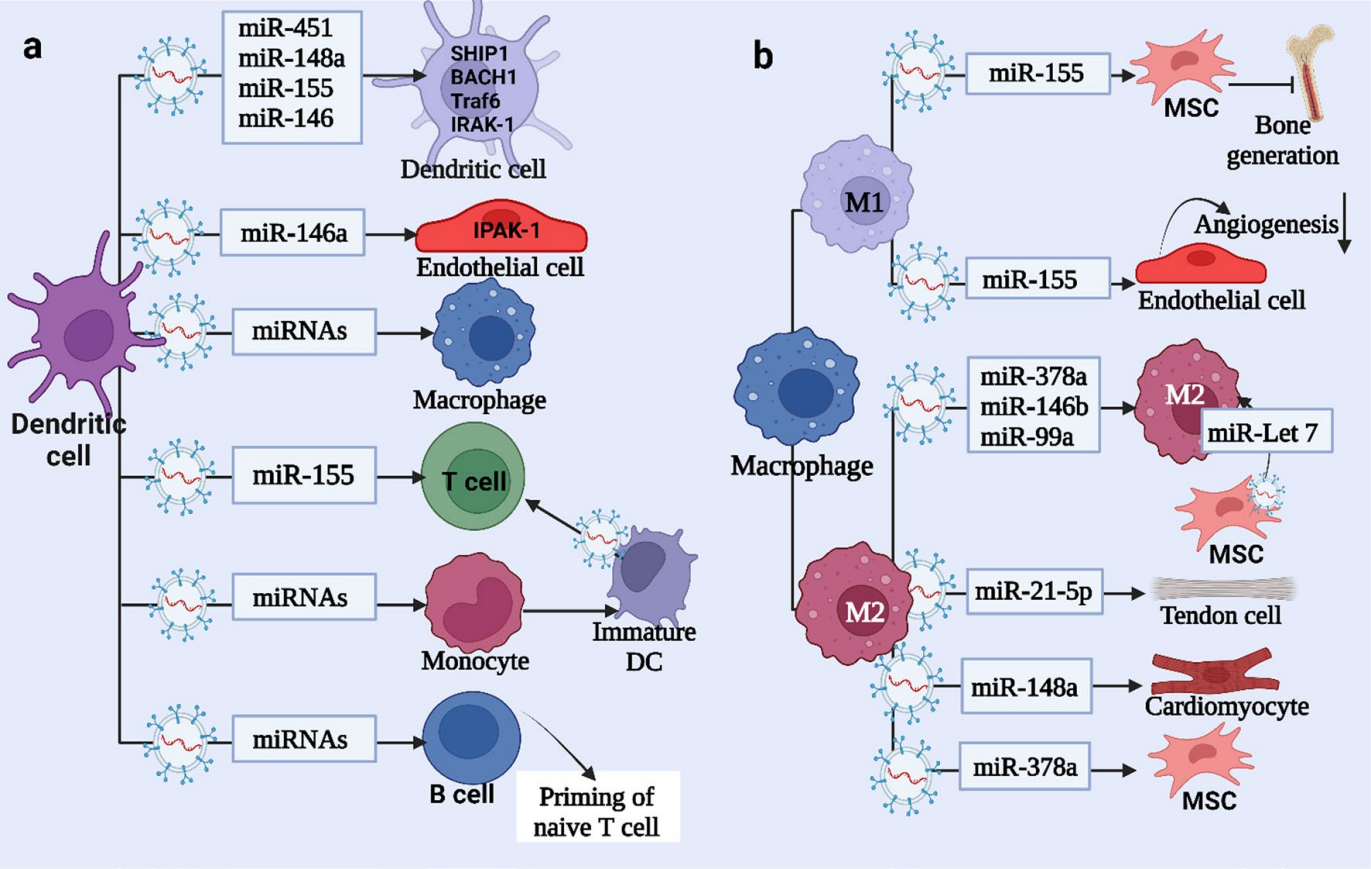
**a** Dendritic cells (DCs) communicate with other DCs through the transfer of exosomal miRNAs, specifically miR-451, miR-148a, miR-155, and miR-146. Monocytes can differentiate into immature DCs, which then activate T cells upon receiving these miRNAs from DCs. **b** Macrophages play a role in regulating various cell types within the body through the transfer of exosomal miRNAs. For instance, M1-derived exosomal miR-155 regulates mesenchymal stem cells (MSCs) and M1-derived exosomal miRNAs inhibit bone generation, while M1-derived exosomal miR-148a regulates angiogenesis in endothelial cells. On the other hand, M2-derived exosomal miRNAs, such as miR-378a, promote bone generation. Additionally, miR-148a induces wound healing in cardiomyocytes, and miR-21-5p stimulates fibrinogenesis in tendon cells

Exosomes mediate communication between DCs as well as between other cells and DCs. For instance, when DCs receive exosomal miRNA-155 of tumor cells, leading to antigen triggering. Also, in the plasma of patients with systemic lupus erythematosus (SLE) activation of plasma cell-like DC and production of proinflammatory cytokines and IFN-α can be induced by TLR7 endogenous ligands within exosomal delivered miRNA. Therefore, miRNAs may represent new pathogenic mediators of autoimmune reactions as well as possible therapeutic targets for type I interferon-mediated illnesses [40]. When immature and mature DCs differentiated into a tolerogenic DC population by endotoxin-treated mesenchymal stem cell (MSC)-derived exosomes, resulting in a dropping in the number of DCs surface markers and proliferation of lymphocytes and release of IL-6, and increasing IL-10, TGF-β releasing. However, the limited targeting and specificity of exosome administration severely limit its usefulness. As a result, Li et al. developed an RNA delivery system based on MSC-derived exosomes capable of targeting particular DC binding and increasing HLA-G expression, with HLA-G inhibiting DC-triggered allogeneic T cell proliferation and eliciting long-term immunological tolerance [41]. Exosomal miR-21-5p and miR-223-3p derived mesenchymal stem cells (MSC) reduce migration and prevent maturation of DCs respectively by acting on the CD83 gene [42]. Exosomes from various origins can thus control DC development, maturation, and function, and they may be significant regulators of DC-induced immunological responses (Fig. 2a) and (Table 1).

**Table 1:** The role of exosomal miRNAs mediating signal transduction between immune cells

MiRNAs	The source of miRNAs	The effect of miRNAs
miR-155 miR-146a miR-148a miR-451	DCs	Modify the target genes SHIP1, BACH1, Traf6, and IRAK-1 on DCs [38]
miRNA-155	Tumor cell	Cause antigen triggering [40]
miR-21-5p miR-223-3p	MSCs	Reduce migration and prevent maturation of DCs [42]
miR-let7	MSCs	Decrease the macrophage infiltration in the plaque [46]
miR-155	M1	Reduces the MSC osteogenic differentiation [47]
miR-378	M2	Increase MSC osteoinductive gene expression [47]
miR-21-5p	M	Enhance fibrogenesis in tendons [48]
miR-222	Breast cancer cell	Macrophage polarization to M2 and tumor progression [158]
miR-23a	HCC	Suppress T-cell function, and increase phosphorylated AKT and PD-L1 expression in macrophages [53]
miRNA-335	T cells	Downregulates translation of SOX-4 mRNA in APCs [62]
mmu-miR-20a-5p mmu-miR-25-3p mmu-miR-155-3p	T cells	Causes modulation of mRNA targets in B cells [63]
miR-142-3p miR-142-5p miR-155	T cells	Induce apoptosis of B cells in type 1 diabetes [64]
Let-7d	Tregs	Decrease INF-γ level produced by Th1 [65]
miR-150-5p miR-142-3P	Tregs	Causes tolerogenic phenotype of DCs, with increased IL-10 and reduced [67]
hsa-miR-7-5P	Sepsis exosomes	Has an antiapoptotic effect on T lymphocytes [68]
hsa-miR-24-3p hsa-miR-891a hsa-miR-106a-5p hsa-miR-20a-5p hsa-miR-1908	NPC	Mediate T-cell dysfunction [69]
miR-125a-3p	MSCs	Suppress the differentiation of effector T cells [42]
miR-10b-5p miR-92a-3p miR-155-5p	NKs	Th1 polarization and IFN-γ and IL-2 production [70]
miR-122 let-7b miR-206	Hepatocyte	Upregulate BAFF expression in macrophages [73]
miRNA-223	Mast cell	Increase the permeability of intestinal epithelial [74]
miR-409-3P	Mast cell	Causes of microglial migration [75]
miR-490	Mast cell	Inhibit tumor metastasis in HCC [76]
miR-142-3p	Neutrophil	Down-regulates PKCα in macrophages [85]
miR-4466	Nicotine-induced N2-neutrophil	Promotes tumor cell metabolic switching [86]
hsa-miR-218-5p hsa-miR-144-3p	Neutrophil	Differentiate between anti-scl70 (-) and anti-scl70 ( +) groups [87]
miR-122-5p	Neutrophil	Increase the permeability of BMECs [88]
miR-30d-5p	Neutrophil	Induce macrophage M1 polarization and primed macrophage pyroptosis [89]
miR-223	Neutrophil	Suppress the inflammasome activation and the canonical NF-κB pathway [90]

### Macrophages

Macrophages act against pathogens and boost wound healing throughout the body, as part of the innate immune system. They are found in both lymphoid and non-lymphoid tissues. Macrophages are categorized based on their basic function and activation. Macrophages can be classified as traditionally activated (M1) macrophages (known as pro-inflammatory), wound-healing macrophages (also known as alternatively activated or anti-inflammatory (M2) macrophages), and regulatory macrophages (Mregs). The M2 macrophages are further sub-categorized into M2a, M2b, M2c, and M2d subtypes. Therefore, M2 macrophage-derived exosomes can be used as a therapy to decrease pro-inflammatory macrophage phenotypes, that most abundant in several diseases such as obesity and atherosclerosis [43]. Ruibing Yang et al. studied the roles of exosomes derived from M2a, M2b, and M2c macrophage phenotypes in colitis triggered by dextran sulfate sodium (DSS). They found that M2b macrophage-derived exosomes significantly reduced the severity of colitis in mice induced by DDS. This is due to M2b macrophage-derived exosomes increasing the splenic number of T regulatory (Treg) cells and the level of IL-4 in mice, while the levels of major cytokines linked to colitis (IL-1, IL-6, IL-7A) were dramatically decreased [44].

Laura Bouchareychas et al. found that polarization of exosomes derived from bone marrow-derived macrophage-derived with IL-4 causes inflammation suppression by targeting tumor necrosis factor-alpha (TNF-α) and nuclear factor kappa B (NF-κB) through miR-146b, miR-99a, and miR-378a in the recipient macrophage [45]. Mesenchymal stem cells (MSCs)-derived exosomes have the ability to improve atherosclerosis in APOE^−/−^ mice by decreasing the macrophage infiltration in the plaque through the miR-let7/ IGF2BP1/PTEN pathway. Further, MSCs-exosomes increased M2 macrophage polarization in the plaque via the miR- let7/HMGA2/NF-κB pathway [46]. It was found that miR-155 in M1 macrophage EV reduces the MSC osteogenic differentiation, while miR-378 in M2 macrophage EV increased MSC osteoinductive gene expression. This confirms the role of M2 macrophage in the bone healing process [47]. Also, some studies showed that miR-21-5p in macrophage-derived exosomes enhanced fibrogenesis in tendons through the change in the Smad7 signaling pathway after injection into mice. In cardiomyocytes, exosomal miR-148a of M2 macrophage modulates the transcription factor thioredoxin-interacting protein (TXNIP) to allow better response to myocardial injury, which is essential to myocardial survival [48].

There are numerous pathways by which tumor-associated macrophages (TAMs) contribute to tumor production, development, metastasis, and immune escape. In tumor cells, exosomes are able to modulate immune responses. Exosomes derived from tumors can induce macrophage polarization, which may result in the activation of anti-inflammatory pro-tumorigenic M2 immune cells or anti-inflammatory antitumorigenic M1 immune cells, alter the M1/M2 ratio in the tumor microenvironment, and enhance tumor growth [49]. Adriamycin-resistant breast cancer cells produce exosomal miR-222, which targets Phosphatase and Tensin Homolog (PTEN) gene, and activates the Akt pathway, leading to macrophage polarization to M2 and tumor progression [50]. Oxidative phosphorylation increases in macrophages in response to let-7a exosomal miR released by the hypoxic tumor cells, leading to the promotion of M2 macrophages and suppression of insulin-Akt-mammalian target of rapamycin (mTOR) signaling pathway [51, 52] (Fig. 2b) and (Table 1).

The exosomal miR-23a-PTEN-AKT pathway could suppress T-cell function via an endoplasmic reticulum stress response in hepatocellular carcinoma cells (HCC) by releasing exosomal miR-23a-3p and upregulating PD-L1 expression in macrophages, according to Liu et al. [53]. Previous studies showed that exosomes have a pivotal role in the polarization of macrophages from one phenotype to another. Holder et al. demonstrated that the human placenta internalizes secreted exosomes from macrophages via the clathrin-mediated endocytosis process. This results in the modulation of proinflammatory cytokine production in the placenta, including IL-6, IL-8, and IL-10. In this way, maternal immune cells communicate with the placenta via exosomes, which may facilitate responses to maternal inflammation and infection, thereby preventing harm to the fetus [54].

### T lymphocytes

T cells have a pivotal role in the adaptive immune system by protecting against infection and cancer, but they are also involved in many immunological diseases. T lymphocytes have different types; the first type is the naïve T cell that matured and is released by the thymus but has not yet encountered its corresponding antigen, so it is called in the stage between maturity and activation. Each naïve T cell has a particular T cell receptor that identifies a specific antigen [55]. The second type is CD8 + T cells (cytotoxic T cells), which have the ability to attack infected cells and kill them using granule sacs that contain digestive enzymes. The third type is CD4 + T cells (helper T cells) which activate CD8 + T cells, macrophages, and increment antibody production by B cell lymphocytes, this process is called immunological response [56]. The fourth type is Regulatory T cells (Tregs) which lower the immune responses when a highly active response is no longer required by monitoring B and T cell functions. The fifth type is Natural Killer T cells which discriminate between infected and malignant cells and target cells that lack molecular markers that identify them as bodily cells. The last one is Memory T cells which give lifelong protection against some diseases by protecting against previously encountered antigens [57].

Exosomes have an immunomodulatory effect on lymphocytes such as regulation of their proliferation, activation, chemotaxis, differentiation, and mediation of diseases. CD8 + T cells induced by tumor-derived exosomes develop a suppressor phenotype (SP), which may act synergistically with exosomal proteins and RNA, making immunosuppressive exosomes a potential therapeutic target [58]. Exosomes derived from Pancreatic cancer (PC) are taken up by T lymphocytes, resulting in a change of gene expression and T lymphocytes exhibit cytotoxic activity after exosome treatment. This is due to PC-derived exosomes that induce Endoplasmic Reticulum (ER) stress-mediated apoptosis of T lymphocytes through p38 MAPK [59, 60]. When a thyroid cell-derived exosome containing TPO, HSP60, and MHC-II has exposed to IFN-γ activated dendritic cells in vitro, causes releasing of proinflammatory cytokines IFN-g, IL-17A, and IL-22 by CD4 + T lymphocytes, while inhibiting IL-4, IL-10, and TGF-b1 expression and release, ultimately suppressing thyroiditis [61].

Exosomes have a critical role in cellular communication during immune synapsis (IS) via the transfer of miRNA. María Mittelbrunn et.al. found that transfer of miRNA-335 of T cells-derived exosome to APCs, in a unidirectional manner, during immune synapses downregulates translation of SRY-Box Transcription Factor 4 mRNA (SOX-4 mRNA), also, exosomal miR-92a level increase in Raji cells after conjugation with T lymphoblasts [62]. Lola Fernández-Messina et.al found that directional transfer of T-cell exosomal miRNA (mmu-miR-20a-5p, mmu-miR-25-3p, and mmu-miR-155-3p) to the B-cell upon immune synapse resulting in B cell function control, proliferation, survival, regulation, and antibody production, by targeting BIM and PTEN [63]. Sometimes T cell exosomes have a cytotoxic effect, for example, Claudine Guay et. al. discovered the transfer of exosomal miRNA (miR-142-3p, miR-142-5p, and miR-155) released by T cells to β cell, insulin-secreting cells, induce apoptosis in type 1 diabetes. This is due to the induction of Ccl2, Ccl7, and Cxcl10 in the pancreatic β cells [64].

**Fig. 3:**
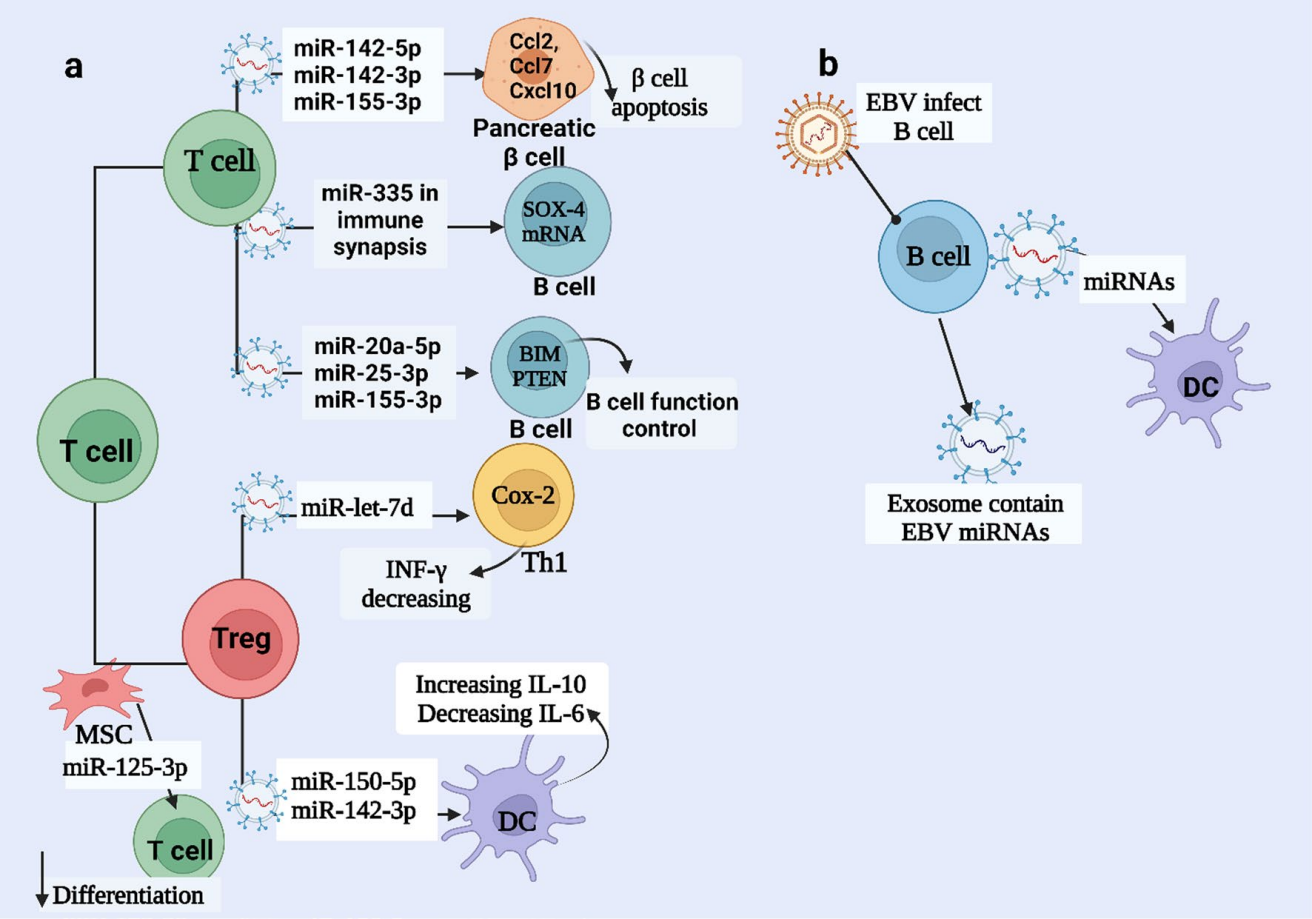
Immunomodulatory Effects of Exosomal miRNA on Lymphocytes. **a** Exosomal miRNA-derived T cells: miR-150-5p and miR-142-3p increase the level of IL-10 and decrease IL-6 production by dendritic cells (DCs). Treg-derived miR-let-7d regulates Th1 cells, and miRNA derived from T cells also regulates B cell functions. **b** Exosomal miRNA-derived B cells influence the function of DCs, especially when the B cells are infected with Epstein-Barr virus (EBV). These infected B cells secrete exosomes containing EBV-derived miRNAs

Some immune cells, including T helper 1 (Th1) cells, were suppressed by transferring miRNAs of Treg cells, which inhibited the proliferation and release of cytokines by Th1 cells. In colitis and autoimmunity, they found that Treg-cell-derived Let-7d-containing exosomes target Cox-2 in Th1 cells leading to decreasing INF-γ, Therefore, lethal Th1-cell-mediated inflammation is prevented [65, 66]. Also, Tregs produce interleukin-35 + EVs that act in trans on bystander T cells, causing exhaustion and suppressing immunity. Transferring of exosomal miR-150-5p and miR-142-3P derived Tregs cells to DCs causes tolerogenic phenotype, with increased IL-10 and reduced IL-6 secretion [67]. Deng, J.-n. et. al. found that hsa-miR-7-5P derived sepsis exosomes have an antiapoptotic effect on T lymphocytes by targeting the proapoptotic gene Bad in the cGMP-PKG signaling pathway [68]. Exosomal miRNA (hsa-miR-24-3p, hsa-miR-891a, hsa-miR-106a-5p, hsa-miR-20a-5p, and hsa-miR-1908) derived-nasopharyngeal carcinoma (NPC) mediate T-cell dysfunction by targeting the down-regulation of the MAPK1 and JAK/STAT pathways [69]. Exosomal miR-125a-3p derived MSC can suppress the differentiation of effector T cells (CD4 + and CD8 +), and improve the survival of CD4 + , CD25 + , Foxp3 + Tregs [42]. Natural killer cell secrets exosomal miR-10b-5p, miR-92a-3p, and miR-155-5p, which target GATA3, and TBX21 leading to Th1 polarization and IFN-γ and IL-2 production [70] (Fig. 3a) and (Table 1).

### B lymphocyte

B cells play an important role in the humoral immunity component of the adaptive immune system. It has many functional roles such as producing antibodies, presenting antigens (antigen-presenting cells (APCs)), and secreting cytokines. B cells differ from T cells that have receptors on their cell membrane known as B cell receptors (BCRs). Through very specific BCRs, B cells bind to the foreign antigens and produce antibodies. Saunderson et al. found that B cells secrete exosomes upon CD40/IL-4 triggering. Exosomes-derived B cell express on their surface high levels of MHC class I, MHC class II, and CD45RA (B220), also, the BCR complex which contains surface Ig, CD19, and the tetraspanins CD9 and CD81 [71]. It is important to note that miRNAs found in exosomes are not always derived from the host genome. Pegtel et al. found that viral (EBV) miRNA secreted by EBV-infected B cells are transferred, by exosomes, to and act in noninfected recipient B cells. These EBV-miRNAs are functional by repression of their target genes including CXCL11/ITAC [72]. Liao et al. found that hepatocyte-derived exosomal miRNA (miR-122, let-7b, and miR-206) could upregulate B-cell activating factor (BAFF) expression in macrophages through Toll-Like Receptor 7 Polymorphism (TLR7) activation [73] (Fig. 3b).

## Mast cells

Mast cell, also known as mastocyte or labrocyte, is a part of the immune and neuroimmune systems, it has many functional roles in allergy (contains a large amount of histamine and heparin granules), anaphylaxis, wound healing, vascular permeability in tumors of brain, and angiogenesis. Exosomes derived from MCs are capable of activating B and T lymphocytes to coordinate pro- and anti-inflammatory processes. Li et al. showed that exosomal miRNA-223-derived mast cells (MCs) increased the permeability of intestinal epithelial cells and destroyed the function of intestinal barrier proteins such as tight junction proteins 1 (TJP1, ZO-1), Occludin (OCLN), Claudin 8 (CLDN8) [74]. Inflammation of the central nervous system (CNS) is a response to external stimuli, including surgery, infection, and toxins, that reveals itself partly in the activation of microglia and the release of proinflammatory cytokines. Hu, L. et.al. showed that exosomal miR-409-3P derived-MCs have a role in neuroinflammation by activation of its target, Nr4a2, in the NF-κB pathway resulting in migration of microglial [75].

**Fig. 4:**
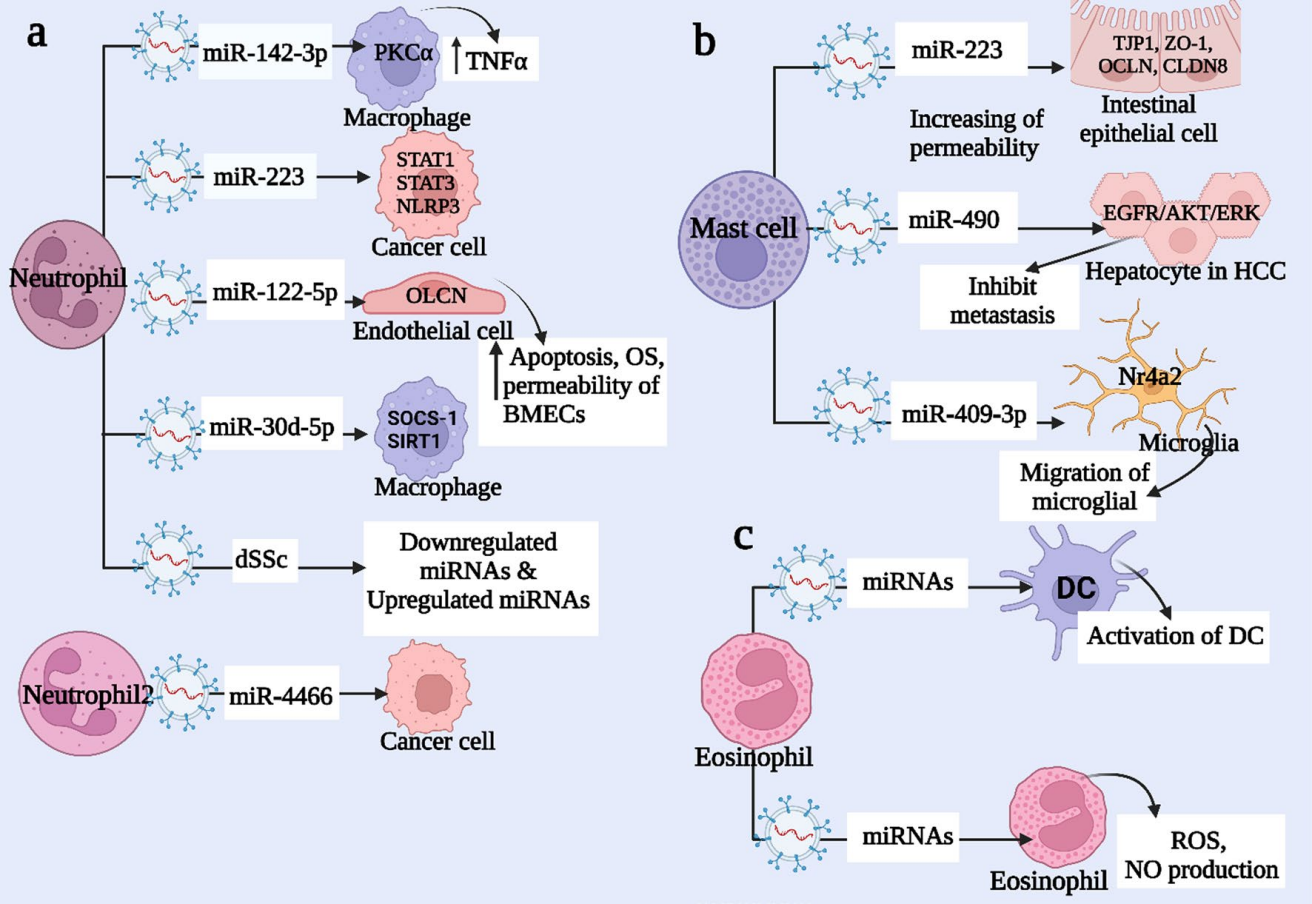
miRNA and Polymorphonuclear Cells: **a** Neutrophils communicate with different cells through exosomal miRNA, which affects their functions. For example, miR-142-3p targets PKCA in macrophages, leading to an increase in TNFα production. In the case of diffuse systemic sclerosis (dSSc), neutrophils secrete various exosomal miRNAs that are either downregulated or upregulated. **b** Mast cell-derived exosomes modulate the function of other cells. For instance, miR-409-3p derived from mast cells promotes microglial migration by targeting Nr4a2. Additionally, miR-223 derived from mast cells increases the permeability of intestinal epithelial cells by targeting TJP1, ZO-1, OCLN, and CLDN8. Lastly, miR-490 derived from mast cells inhibits metastasis in hepatocellular carcinoma by targeting the EGFR/AKT/ERK pathway. **c** Eosinophils release exosomes that can be taken up by other eosinophils, resulting in the production of reactive oxygen species (ROS) and nitric oxide (NO). Furthermore, miRNAs derived from eosinophils can activate dendritic cells (DCs)

In addition, MCs-derived exosomes have a role in the migration and invasion of hepatocellular carcinoma (HCC) cells. It was found that exosomal miR-490 derived-MCs can inhibit tumor metastasis in HCC by dropping the activity of the EGFR/AKT/ERK1/2 pathway [76]. For regulation of tumor cell proliferation, it was shown that tryptase secreted from MCs binds to DNA present on the surface of melanoma cell-derived exosomes, resulting in degradation of lamin B (cause nuclear remodeling), hnRNP A2/B1, suppression of SNORA55 and RNU-2 expression, upregulation of MIR16–2 expressions, and downregulation of EGR1, all of these affect the gene expression and decreased proliferation [77, 78] (Fig. 4b) and (Table 1).

## Eosinophils

Eosinophils are one of the immune system components, they play a role in fighting viral infections and helminth, also, implicated in antigen presentation to cells. Furthermore, eosinophils are important mediators of allergic responses and asthma pathogenesis. Exosomes have an effective role in the pathology of asthma as well as other inflammatory diseases. The exosomes-derived eosinophils are able to stimulate eosinophils for the production of reactive oxygen species (ROS) and nitric oxide (NO) this process is called the autocrine effect [79] (Fig. 4c). Mazzeo et al. was the first group showed that eosinophils have MVBs and release exosomes which raised in asthmatic patients [80]. They described how the eosinophil exosomes damage the airways in the case of asthma disease. Bronchial smooth muscle cells (BSMC) and small airway epithelial cells (SAEC) structurally remodel the airways and regulate respiratory function and immunity. Exosomes derived- asthmatic eosinophils change the behavior of SAEC by increasing their apoptosis, decreasing their wound-healing capacity, and increasing their expression of CCL26, TNF-α, and POSTN. Furthermore, asthmatic exosomes modify the performance of BSMCs, increasing their proliferation and upregulating CCR3 and VEGFA gene expression through ERK1/2-phosphorylation [81].

The differential expression of 14 miRNAs between asthmatic and healthy eosinophils was validated by previous studies. There are multiple genes associated with inflammation are directly or indirectly regulated by miRNAs such as miR-16 downregulates (tumor necrosis factor-alpha) TNFα; the miR-17 ~ 92 cluster modifies PTEN; miR-155 alters PU.1, c-Maf, and SOCS1; and miR-146a regulates TRAF6 and IRAK1. There are other miRNAs associated with the pathology of asthma and allergies, such as miR-21, which targets IL-12p35 in steroid-resistant asthma; miR-1248, which targets IL-5; and miR-15a, which inhibits VEGFA in CD4 + cells. miR-185-5p expression is a good biomarker of asthma disease severity, as it has a role in the sensitivity of corticosteroids by suppressing the mammalian target of rapamycin complex signaling pathway (mTORC) so miR-185-5p can restore sensitivity in CEM-C1 cells which are steroid-resistant. Also, miR-185-5p has a role in cell growth by targeting CDC42 and RHOA directly and VEGFA indirectly. Finally, miR-185 is involved in calcium signaling by targeting NFAT and CaMKII in myocardial cells during cardiac hypertrophy [82]. Gao et al., showed that exosomal miR-100-5p derived from human umbilical cord mesenchymal stem cell (hUCMSC) has an important role in atherosclerosis alleviation in ApoE^−/−^ mice by repressing cell migration, inducing apoptosis, and preventing inflammatory response in eosinophils through the FZD5/Wnt/β-catenin pathway [83].

## Neutrophil

Neutrophils (also called neutrocytes, heterophils, or polymorphonuclear leukocytes) are the most common form of granulocyte, accounting for 40 to 70% of all white blood cells in humans. It is estimated that 40 to 70% of human granulocyte white blood cells are neutrophils (polymorphonuclear leukocytes). They have an essential role in the innate immune system, they are present in blood circulation as phagocytes. The first (acute) phase of inflammation occurs when neutrophils migrate into the inflammatory site, especially when bacteria infect, environmental toxins are present, and certain cancers are present. Neutrophils move to blood arteries and then into interstitial space following chemical cues such as interleukin-8 (IL-8), C5a, fMLP, Leukotriene B4, and H_2_O_2_ this process is called chemotaxis. In response to the immune complex, neutrophils release multiple molecules outside the cells, such as proteases, cytokines, reactive oxygen species, and exosomes. Neutrophils also collaborate with macrophages, dendritic cells, B and T cells, and natural killer cells (NKs) to regulate the immune response by producing IFN-γ. Neutrophil-derived EXOs (neutrophil EXOs) also play important roles in immune responses. The neutrophil EXOs are capable of modulating immune responses, increasing smooth muscle cell proliferation in the lungs, and promoting reorganization of the airways in asthmatic patients [84]. Linhares-Lacerda et al. exhibited that miR-142-3p is transported from neutrophil extracellular traps (NETosis) to macrophages where it down-regulates protein kinase C alpha (PKCα) resulting in the neutralization of the excessive production of TNFα. Also, Different methods of NETosis induce different expression levels of miRNAs [85].

Recent studies have revealed that neutrophils can also functionally polarize, determining selective activity patterns related to different diseases. Neutrophils are classified into two well-defined types, known as pro-inflammatory (immunostimulating) N1s and suppressor (immunosuppressive) N2s. Tyagi et al. showed that the SOX2/CPT1A axis promotes tumor cell metabolic switching via exosomal miR-4466 secreted from nicotine-induced N2-neutrophils [86]. Li et al. identified the profile of exosomal miRNAs-derived neutrophils in diffuse cutaneous systemic sclerosis (dSSc). This profile includes downregulated miRNAs (such as hsa-miR-299-3p, hsa-miR-1323, hsa-miR-520a-3p, hsa-miR-512-3p, and hsa-miR-4755-3p) and upregulated miRNAs (such as hsa-miR-3614-5p, hsa-miR-323a-3p, hsa-miR-95-3p, hsa-miR-1260b, hsa-miR-1248, hsa-miR-1260a, hsa-miR-1268a, and hsa-miR-218-5p). The target genes of exosomal miRNAs-derived neutrophils were enriched in Wnt and AMPK signaling pathways. The expression of hsa-miR-218-5p and hsa-miR-144-3p were significantly different between anti-scl70 (−) and anti-scl70 ( +) groups from dysregulated miRNAs [87].

Occludin (OCLN) coded transmembrane proteins play a critical regulatory role in protein tight junction formation and cell permeability barrier and are essential components of the blood–brain barrier. Li et al. found that exosomal miR-122-5p derived lipopolysaccharide (LPS)-induced neutrophils promote oxidative stress, and apoptosis and increase the permeability of brain microvascular endothelial cells (BMECs) by the down-regulation of OLCN expression [88]. Polymorphonuclear neutrophils (PMNs) play a crucial role in the pathogenesis of sepsis-related acute lung injury (ALI) or acute respiratory distress syndrome (ARDS). Jiao et al. It was found that exosomal miR-30d-5p derived PMNs induce macrophage M1 polarization and primed macrophage pyroptosis by activating NF-kB signaling by targeting SOCS-1 and SIRT1 [89]. Exosomal miR-223-derived neutrophil represses the canonical NF-κB pathway by downregulating the expression of components in the signaling transduction pathway [90]. Furthermore, exosomal miR-223 derived from neutrophils suppresses inflammasome activation and the production of inflammatory cytokines by directly targeting STAT1, STAT3, and NLRP3. In cancer cells, it also regulates genes involved in cell proliferation, survival, differentiation, immune evasion, cell adhesion, and migration [90] (Fig. 4a) and (Table 1).

## Tumor microenvironment

Tumors are heterogeneous tissues of cancer cells, infiltrating and resident host cells, secreted factors, and extracellular matrix. The growth and progression of tumors are facilitated by the molecular, cellular, and physical changes that tumor cells cause within their host tissues. The tumor microenvironment (TME), known as the cancer microenvironment, is a complicated and continuously evolving entity. The TME components are heterogeneous and depend on the type of tumor, but the most common include endothelial cells, pericytes, fibroblasts, immune cells, stromal cells, blood vessels, and extracellular matrix. Truffi et al. suggested that the "tumor microenvironment is not merely a mute bystander, but rather an active supporter of cancer growth" [91]. In the early stages of tumor development, cancer cells, and TME components develop a dynamic relationship that enables the survival, infiltration, and metastatic spread of cancer cells. As a means of restoring oxygen and nutrient supply and removing metabolic waste, the TME promotes angiogenesis to overcome a hypoxic and acidic microenvironment [92]. As tumors grow, they become infiltrated with diverse immune cells, including adaptive (including T cells, B cells, and natural killer (NK) cells) and innate (macrophages, neutrophils, and dendritic cells) cells, which can both promote tumor growth and inhibit it [93]. The most prevalent mechanism of tumor formation is chronic infection-induced persistent inflammation in the case of colorectal, hepatocellular, and cervical cancers.

As mentioned before, TME is characterized by hypoxia compared to the normal internal environment, this is due to the release of matrix metalloproteinases (MMPs), hypoxia-inducible factor-1α (HIF-1α), vascular endothelial growth factor (VEGF) and other stimulating factors [94]. TME remodeling creates a niche for tumor cells to interact with neighboring fibroblasts, endothelial cells, and immune cells [95]. This interaction triggers a multitude of biological processes for tumor progression, including appreciation, migration, angiogenesis, immunosuppression, and drug resistance [96]. miRNAs regulate diverse biological functions through the regulation of gene expression, including proliferation, apoptosis, differentiation, migration, invasion, and drug resistance. Changes in genetics or epigenetics can lead to abnormal expression of miRNAs in cancer cells, resulting in abnormal gene expression [97, 98]. miRNAs suppress gene expression at the protein level by interacting with complementary target mRNA at 6–7 bases. Previous studies showed that miRNAs can trigger the development of TMEs and biological alterations by acting as oncogenes. Normal fibroblasts (NFs) within the tumor microenvironment (TME) undergo transformation into cancer-associated fibroblasts (CAFs) through the actions of miR-9, miR-200 s, miR-526b, and miR-655 which enhance angiogenesis and lymphangiogenesis within the TME. Additionally, miR-340-5p and miR-561 contribute to the promotion of an immunosuppressive microenvironment. Exosomes, which transport proteins, metabolites, and nucleic acids (including miRNAs), play a crucial role in mediating communication between tumor cells and the TME. Consequently, exosomes are the primary modulators responsible for the heterogeneity of the TME and exert significant influence over tumor development.

### TME and immune cells

Over the years, there has been a growing recognition of the importance of communication between cancer cells and immune cells, leading to the inclusion of this interaction as one of the emerging hallmarks of cancer by 2011 [99]. It is now well-established that the tumor microenvironment (TME) comprises cancer cells and various immune cells, stromal cells, endothelial cells, and cancer-associated fibroblasts. Cancer cells employ diverse mechanisms to evade immune surveillance and evade destruction. Consequently, several immunotherapy approaches have been developed and applied in clinical practice over the past decades, aiming to counteract these immune evasion mechanisms. Unlike traditional chemotherapy, immunotherapy primarily leverages the immune cells within or outside the TME to selectively recognize and attack cancer cells. This theoretically grants immunotherapy higher specificity and lower side effects [100].

The immune cells within the tumor microenvironment (TME) exhibit diverse functions. For instance, tumor-antagonizing immune cells, such as effector T cells, kill target cells through granule exocytosis and FasL-mediated apoptosis induction. They also secrete IFN-γ and TNFα, which induce cytotoxicity in cancer cells. NK cells contribute to the tumor-killing response by releasing perforin and granzymes to induce apoptosis in target cells. They also secrete pro-inflammatory cytokines and chemokines (such as IFN-γ, TNF, IL-6, GM-CSF, and CCL5) to promote anti-tumor activity [101]. DCs present antigens, provide costimulatory signals for T-cell activation, and interact with NK and B cells [102]. M1-polarized macrophages generate pro-inflammatory cytokines and reactive oxygen/nitrogen species to eliminate tumor cells [103], while N1-polarized neutrophils release granules containing antimicrobial and cytotoxic compounds to destroy cancerous cells. Additionally, these immune cells secrete cytokines and chemokines to attract other cells with anti-tumor activity [104].

On the other hand, immune cells that promote tumor growth include regulatory T cells, which hinder the effective response of effector T cells against cancer cells. MDSCs (myeloid-derived suppressor cells) enhance angiogenesis by producing MMP9, prokineticin 2, and VEGF, promoting the migration of cancer cells toward endothelial cells and facilitating metastasis [105]. There is controversy surrounding the role of B cells. On one hand, they can produce cytokines that coordinate with cytotoxic T lymphocyte (CTL) activity and act as potent antigen-presenting cells (APCs). However, B cells may also possess a protumorigenic potential by producing cytokines that recruit MDSCs and enhance angiogenesis [106]. They can inhibit CTL function as well.

### TME and exosomal miRNAs

As the tumor progresses, exosomal miRNAs produced by primary tumor cells are transferred to non-malignant cells in the TME to promote heterogeneity [107]. While non-malignant cells can release exosomal miRNAs as a result of changing their biological activities in the TME, non-malignant cells can also secrete miRNAs to further regulate tumor cells [108]. Most previous studies have identified cancer-associated fibroblasts (CAFs), endothelial cells, and immune cells as the stromal cell receptors of cancer-derived exosomal miRNAs. Within the TME, the impact of exosomal miRNAs is particularly evident in the activation of CAFs, which remodel the extracellular matrix (ECM) and promote tumor cell dissemination. In response to exosomal miRNAs, Endothelial cells facilitate the formation of tube networks that enhance tumor cell metabolism and survival. Furthermore, exosomal miRNAs contribute to the invasion of inflammatory cells and aid in immune evasion, thereby promoting tumor colonization and proliferation. Considering these significant effects, exosomal miRNAs play a role in facilitating tumor development within the TME [109, 110].

*Enhancing growth and metastasis through angiogenesis:* Tumor development is highly reliant on cancer cell metabolism. A tumor with irregular blood vessel distribution and abnormal vascular function results in local hypoxia and reduced nutrition supply. Simultaneously, the distance gradient between various vascular beds causes an imbalance in medication distribution and absorption [111]. These vascular network modifications encourage the establishment of an internal microenvironment and intratumoral heterogeneity. The extensive vascular network in TME promotes cancer cell growth and metastasis. Blood vessels in the microenvironment can be modified by exosome-bound miRNAs, which are taken up and used by vascular endothelial cells. Exosomal miRNAs-derived tumor cells have been shown to induce angiogenesis in TME.

For instance, miR-23a, originating from nasopharyngeal carcinoma (NPC), exerts a crucial role in promoting angiogenesis by selectively targeting TSGA10. This targeting facilitates the phosphorylation of p-ERK and modulates the growth, migration, and tube formation of human umbilical vein endothelial cells (HUVECs) [112]. In glioma, both miR-21 and miR-9 induce angiogenesis by activating the VEGF/p-FLK/VEGFR2 and MYC/OCT4 pathways, respectively [113, 114]. Similarly, in TGF-β1 lung cancer, miR-619-5p and miR-142-3p contribute to angiogenesis by inhibiting RCAN1,4 and PDGFR-β, as well as p-SMAD2/3, respectively [115, 116]. This angiogenic phenomenon extends to other miRNAs derived from hepatocellular carcinoma (HCC), such as miR-210-3P, which targets SMAD4 and STAT6, as well as miR-100 derived from mesenchymal stem cells (MSCs), which induces angiogenesis in breast cancer by targeting the mTOR/HIF-1α/VEGF signaling axis [117, 118]. Additionally, miR-126a, derived from myeloid-derived suppressor cells (MDSCs), enhances angiogenesis by activating inflammatory circuits and increasing IL-13 and IL-33 levels [119]. In ovarian cancer, miR-205 promotes angiogenesis by targeting the PTEN/AKT pathway, while miR-141-3p derived from the human ovarian carcinoma cell line SKOV-3 activates the JAK-STAT3 pathway in endothelial cells to induce angiogenesis [120, 121]. Exosomal miR-205-5p from NPC promotes angiogenesis and NPC metastasis by targeting DSC2 to enhance the EGFR/ERK signaling and MMP expressions [122]. Exosomal miR-9-3p from Müller glia cells enhances retinal angiogenesis by targeting the S1P_1_/AKT/VEGFR2 pathway in diabetic retinopathy [123].

**Fig. 5:**
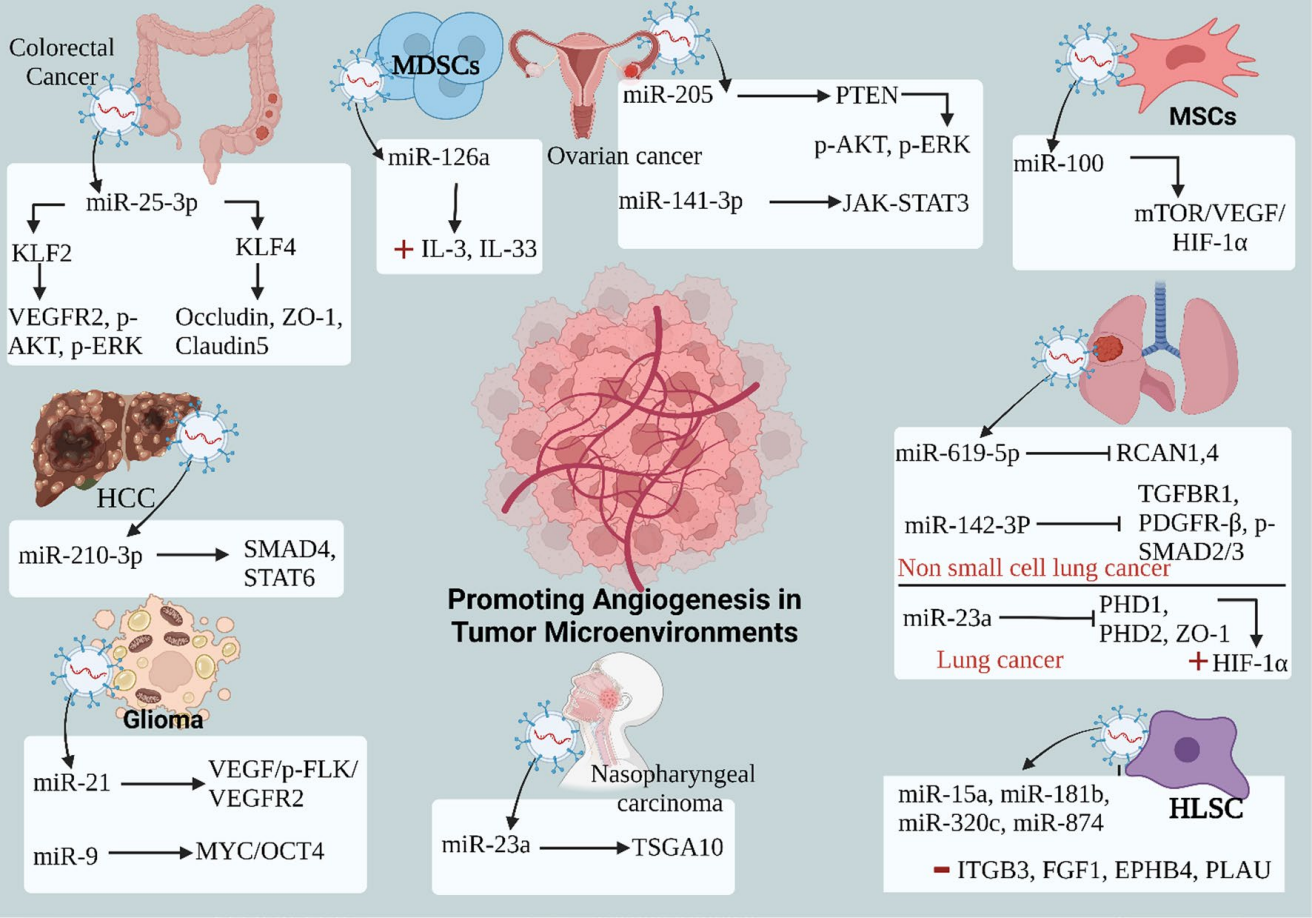
Mechanisms of Exosomal miRNA Regulation of Angiogenesis in Tumor Microenvironments. Exosomal miRNAs taken up by endothelial cells in the tumor microenvironment can affect angiogenesis. For example, in nasopharyngeal carcinoma (NPC), miR-23a-derived exosomes promote angiogenesis by targeting TSGA10 and activating the phosphorylation of p-ERK. In glioma, miR-21 and miR-9 induce angiogenesis by activating the VEGF/p-FLK/VEGFR2 pathway and MYC and OCT4, respectively. In non-small lung cancer, miR-619-5p and miR-142-3p promote angiogenesis by inhibiting RCAN1,4 and PDGFR-β, and p-SMAD2/3, respectively. Similar mechanisms apply to other types of cancer. For instance, in hepatocellular carcinoma (HCC), mesenchymal stem cells (MSCs), myeloid-derived suppressor cells (MDSCs), and ovarian cancer, miRNAs such as miR-210-3p, miR-100, miR-126-a, miR-205, and miR-141-3p promote angiogenesis, while miRNAs derived from hepatic lineage stem cells (HLSC) like miR-15a, miR-181b, miR-874, and miR-320c inhibit angiogenesis. *( +): increase, (−): decrease

Furthermore, exosomal miR-25-3p, originating from colorectal cancer, exhibits the ability to downregulate KLF2 and KLF4 expression. KLF2 engages the VEGFR2/p-Erk/p-Akt pathway, influencing the tube formation ability of HUVECs, whereas KLF4 activates the ZO-1/Occludin/Claudin5 pathway, leading to modifications in the vascular microenvironment, including vascular permeability, adhesion, and the formation of a circular structure in aortic rings [124]. Additionally, miR-23a derived from lung cancer promotes angiogenesis by inhibiting prolyl hydroxylase 1 and 2 (PHD1 and PHD2), resulting in HIF-1α accumulation in endothelial cells and increased permeability of vascular trans-endothelial migration of cancer cells by inhibiting ZO-1 [125]. In contrast, miRNAs derived from human liver stem cells (HLSC), including miR-15a, miR-181b, miR-874, and miR-320c, exert an anti-tumorigenic effect by suppressing tumor angiogenesis through the downregulation of target genes such as ITGB3, FGF1, EPHB4, and PLAU [126]. Also, miR-451a derived from HCC inhibits tube formation and vascular permeability of HUVECs through targeting LP1N1 [127]. Moreover, miR-9 derived from NPC demonstrates anti-angiogenic effects by up-regulating MDK and activating the PDK/Akt signaling pathway, thereby inhibiting the formation of endothelial cells [128]. The mechanisms of inducing angiogenesis by exosomal miRNAs are summarized in (Fig. 5) and (Table 2).

**Table 2:** The effect of exosomal miRNAs in the tumor microenvironment (TME)

miRNAs	The source of miRNAs	The effect of miRNAs & Mechanism of action
miR-21	Glioma	Induce angiogenesis by targeting VEGF/p-FLK/ VEGFR2 signaling pathway [113]
miR-210-3p	HCC	Induce angiogenesis by targeting SMAD4 and STAT6 [117]
miR-142-3p	NSCLC	Induce angiogenesis by inhibiting TGFβR1, PDGFR-β, and p SMAD2/3 [116]
miR-141-3p	Ovarian Cancer	Induce angiogenesis by activating the JAK-STAT3 pathway [121]
miR-23a	NPC	Induce angiogenesis by targeting TSGA10 and modulate the growth, migration, and tube formation of HUVECs [112]
miR-100	MSCs	Induce angiogenesis by targeting the mTOR/HIF-1α/VEGF signaling axis [118]
miR-619-5p	NSCLC	Induce angiogenesis by inhibiting the expression of RCAN1.4 [115]
miR-15a miR-181b miR-320c miR-874	HLSC	Inhibit tumor angiogenesis by downregulation of the target genes including ITGB3, FGF1, EPHB4, and PLAU [126]
miR-205	Ovarian Cancer	Induce angiogenesis by targeting the PTEN/AKT pathway [120]
miR-25-3p	Colorectal Cancer	Induce angiogenesis by targeting KLF2 and KLF4 through the VEGFR2/p-Erk/p-Akt pathway and ZO-1/Occludin/Claudin5 pathway respectively [124]
miR-23a	Lung Cancer	Induce angiogenesis by inhibiting PHD1, PHD2, and ZO-1 and accumulating HIF-1α [125]
miR-9	Glioma	Induce angiogenesis by targeting the MYC/OCT4 pathway [114]
miR-451a	HCC	Inhibit angiogenesis by targeting LPIN1 [127]
miR-9	NPC	Inhibit angiogenesis by regulating MDK and activating the PDK/Akt signaling pathway [128]
miR-126a	MDSCs	Induce angiogenesis by activating inflammatory circuits and increasing IL-13 and IL-33 [119]
miR-212-3p	Pancreatic Cancer	Suppress the immune system by interfering with DCs function by targeting MHC II TF RFXAP resulting in reduced expression of HLA-DR, -DP, and -DQ molecules [139]
miR-203	Pancreatic Cancer	Suppress the immune system by Influence on NKs activation by reducing TLR4, TNF-α, and IL-12 levels in DCs [140]
miR-let-7i	Breast cancer	Suppress the immune system by changing the levels of IL-6, IL-17, IL-1b, TGFβ, SOCS1, KLRK1, IFNγ, and TLR4 [142]
miR-150-5p miR-142-3p	Treg	Suppress the immune system by stimulating a cell-refractory phenotype of DCs, resulting in increased IL-10 and decreased IL-6 levels [141]
miR-let-7d	Treg	Suppress the immune system by inhibiting Th1 cell proliferation and IFNγ secretion [65]
miR-125b	NSCLC	Enhancing the immune system by macrophage repolarization to M1 phenotype [159]
miR-125b-5p	Melanoma	Enhancing the immune system by targeting LIPA and increasing IL-1β, CCL1, CCL2, and CD80 marker’s level of M1 phenotype [5]
miR-21	Head and Neck Cancer	Suppress the immune system by macrophage polarization to M2 phenotype and increasing MRC1 level of M2 [137]
miR-222-3p	EOC	Suppress the immune system by macrophage polarization to M2 phenotype by phosphorylation of STAT3 and downregulating SOCS3 [134]
miR-21-3p miR-125b-5p miR-181d-5p miR940	EOC	Suppress the immune system by TAMs polarization to M2 phenotype [135]
miR-301a-3p	Pancreatic Cancer	Suppress the immune system by M2 phenotype polarization of macrophages by activation of the PTEN/PI3Kγ signaling pathway [160]
miR-1246	Colon Cancer	Suppress the immune system by M2 phenotype polarization of macrophages by increasing IL-10, TGFβ, and MMPs level [136]
miR-155	CLL	Suppress the immune system by reprogramming conventional monocytes into MDSCs by nuclear translocation of NFkB and phosphorylation of STAT1 [144]
miR-10a	Glioblastoma	Suppress the immune system by abnormal differentiation of MDSCs by targeting RORA through the NFκB pathway [143]
miR-21	Glioblastoma	Suppress the immune system by abnormal function of MDSCs by targeting PTEN through the p-STAT3/p-p65/p-Akt pathway [143]
miR-124	Ovarian Cancer	Remodel ECM by differentiating NFs into CAFs by targeting SPHK1 and upregulation α-SMA and FAP [147]
miR-27b-3p miR-214-3p	Myeloma	Remodel ECM by activation of fibroblast marker, α-SMA, and FAP, through targeting the FBXW7 and PTEN/AKT/GSK3 pathways [148]
miR-27a	Gastric Cancer	Remodel ECM by differentiating NFs into CAFs by decreasing the level of CSRP2, and increasing the level of α-SMA [149]
miR-10b	Colorectal Cancer	Remodel ECM by differentiating NFs into CAFs by inhibiting PIK3CA level and PI3K/Akt/mTOR pathway activity, increasing the level of TGF-β and α-SMA [150]
miR-21	HCC	Remodel ECM and enhance angiogenesis by reprogramming hepatic stellate cells (HSCs) into CAFs by targeting PTEN, activating PDK1/Akt signaling, and increasing VEGF, MMP2, MMP9, bFGF, and TGF-β [152]
miR-9	Breast Cancer	Remodel ECM by differentiating NFs into CAFs by affecting MMP1, EFEMP1, and COL1A1 [154]
miR-105	Breast Cancer	Remodel ECM by reprogramming stromal cells by activating MYC signal transduction in CAFs [155]
miRNA-142- 3p	Lung Cancer Cells	Remodel ECM by differentiating NFs into CAFs [116]
miR-155	Melanoma	Remodel ECM and enhance angiogenesis by reprogramming NFs into CAFs by inhibiting SOCS1 and activating the JAK2/STAT3 signaling pathway and increasing levels of VEGFa, FGF2, and MMP9 in fibroblasts [151]
miR‐1249‐5p miR‐6737‐5p miR‐6819‐5p	Colorectal Cancer	Remodel ECM by activating fibroblasts to CAFs by suppressing TP53 expression in fibroblasts [161]
miR-1247	HCC	Remodel ECM by activating β1-integrin–NF-κB signaling in fibroblasts to reprogramme into CAFs by targeting B4GALT3, leading to increasing in IL-6 and IL-8 levels [162]

*Immunomodulatory effect:* Immune cells such as lymphocytes, dendritic cells, and macrophages invade tumor tissues and surrounding locations frequently in the TME. Tumor cells can suppress immune cell maturation and differentiation via numerous signal transduction pathways controlled by exosomal miRNAs, so producing an immunological milieu favorable for tumor development. At the same time, tumor cells frequently secrete metabolic by-products such as lactic acid, nitric oxide, reactive oxygen species, prostaglandins, and arachidonic acid in hypoxia and low nutrient supplies in the microenvironment, leading to the formation of an inflammatory microenvironment. In the tumor microenvironment, tumor cells (outbreak, escape) from immune surveillance due to the changes in the biological activity of numerous immune cells and the generation of inflammatory mediators [129].

Tumor-associated macrophages (TAMs) are essential components of the tumor microenvironment (TME), derived from circulating monocytes recruited to the tumor site. TAMs exhibit a complex and diverse role in tumor progression, displaying both pro-tumorigenic and anti-tumorigenic functions depending on their polarization state and the specific context of the TME. Pro-tumorigenic functions of TAMs involve tumor promotion by secreting growth factors, cytokines, and chemokines that enhance tumor cell proliferation, survival, and migration. They also promote angiogenesis through the secretion of pro-angiogenic factors, facilitating the formation of new blood vessels to support tumor growth and metastasis [130]. Additionally, TAMs contribute to immune suppression by releasing immunosuppressive factors that inhibit the activity of cytotoxic T cells and natural killer (NK) cells [131]. Conversely, TAMs exert anti-tumorigenic effects through tumor surveillance, participating in the recognition and elimination of tumor cells via phagocytosis and antigen presentation, thereby contributing to immune surveillance and tumor control. TAMs are also involved in tissue remodeling and wound healing by facilitating debris clearance and promoting extracellular matrix remodeling. Moreover, TAMs can be polarized towards an anti-tumor phenotype known as M1-like TAMs, which secrete pro-inflammatory cytokines and activate immune responses against the tumor. The polarization state of TAMs is highly influenced by the TME, including signals from tumor cells, stromal cells, and soluble factors present in the microenvironment. The interplay between TAMs and other immune cells, such as T cells and natural killer cells, is crucial in determining the overall immune response against the tumor [128, 132].

From these miRNAs, miR-301a-3p derived from hypoxic pancreatic cancer cells induces M2 polarization of macrophages by targeting PTEN and activating the p-mTOR, p-AKT, and PI3Kγ signaling pathways [133]. Moreover, miR-222-3p from EOC (epithelial ovarian cancer) activates macrophages, leading to the formation of TAMs via the SOCS3/STAT3 pathway [134]. Additionally, miR-21-3p, miR-125b-5P, miR-181D-5p, and miR-940 secreted from hypoxia EOC induce M2 macrophage polarization resulting in tumor progression [135]. Furthermore, M2 polarization induced by miR-21 from human head and neck cancer increases MRC1 of M2, and miR-1246, which are derived from colon cancer increase IL-10, TGFβ, AND MMPs levels [136, 137]. These exosomal miRNAs play a pivotal role in modulating the immune microenvironment by mediating immunosuppression. However, exosomal miR-125-5p derived from melanoma enhances immunity by elevating the levels of M1 phenotype markers IL-1β, CCL1, CCL2, and CD80 through the targeting of LIPA [5]. Conversely, the internalization of exosomal miR-21 by CD14 + human monocytes suppress the expression of the M1 marker while simultaneously enhancing the expression of the M2 marker such as in the case of miR-21 from human head and neck cancer.

**Fig. 6:**
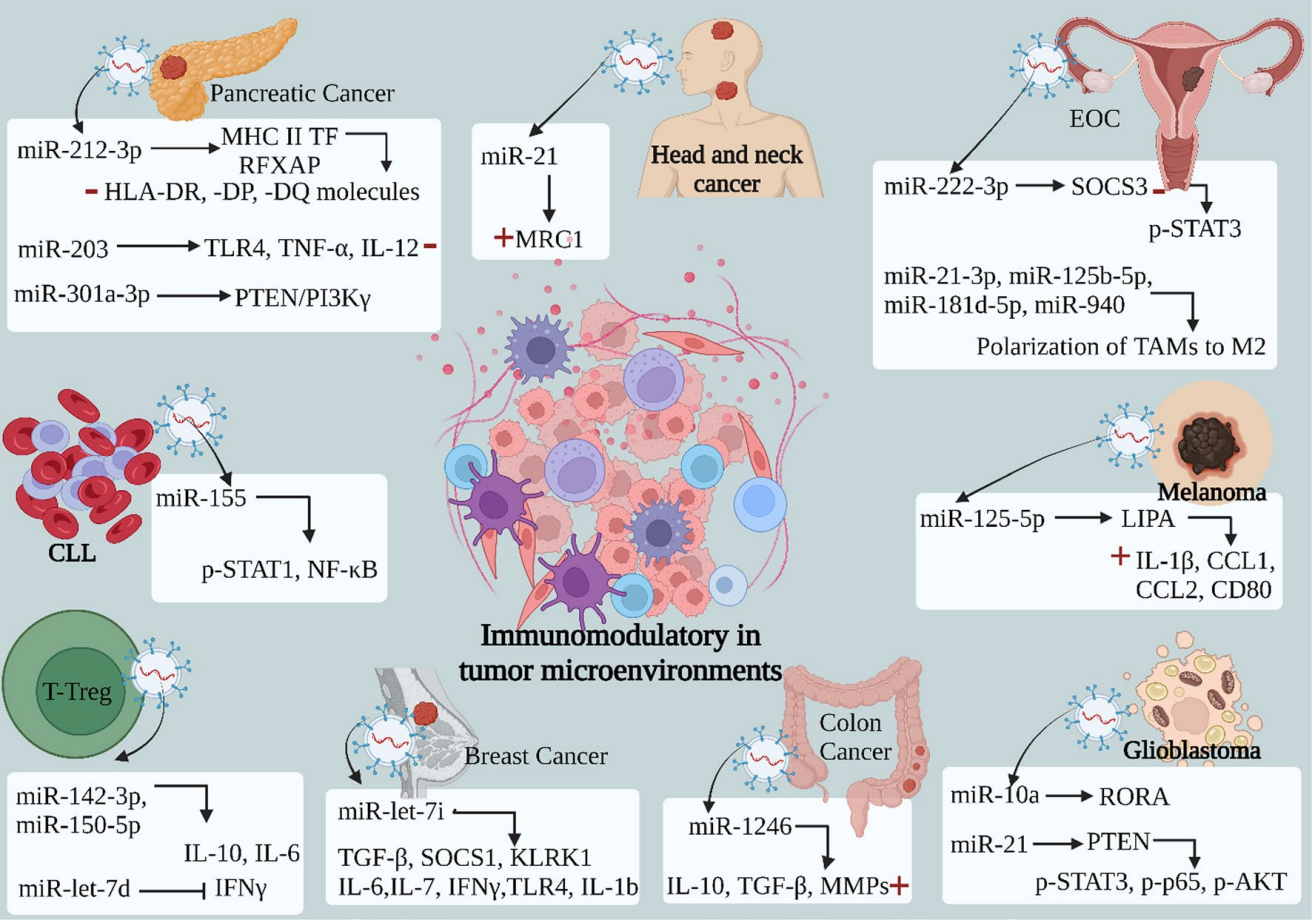
The immunomodulatory effect of exosomal miRNA in tumor microenvironments: Exosomes released by primary tumor cells are absorbed by receptors of the immune cells, wherein the exosomal miR-212-3p derived pancreatic cancer targets MHC II TF RFXAP result in reducing the expression of HLA-DR, -DP, and -DQ molecules which interfere with DCs cells function, miR-203 downregulates TLR4 and downstream cytokines in DCs, and miR-301a-3p induced the M2 polarization of macrophages via targeting PTEN and activation of the p-mTOR, p-AKT, PI3Kγ signaling pathway. In breast cancer, miR-let-7i induces DC maturation by decreasing TGF-β, SOCS1, and KLRK1 levels and increasing IL-6, IL-7, IFNγ, and IL-1b. miR-22-3p derived EOC activates macrophages to a TAM through SOCS3/STAT3 pathway. Also, miR-21-3p, miR-125B-5P, miR-181D-5p, and miR-940 induce M2 macrophage polarization.M2 polarization induced by miR-21 derived human head and neck cancer secrets miR-21 and miR-1246 derived colon cancer. DCs took up miR-142-3p, miR-150-5p derived Treg resulting in induction of a tolerogenic phenotype in these cells, with increased IL-10 and decreased IL-6 production, while miR-let-7d derived Treg inhibit Th1 proliferation and IFNγ. Glioblastoma secretes miR-10a and miR-21, which affect the activation and differentiation of MDSCs by targeting RORA and PTEN, respectively. These exosomal miRNAs modulate the immune microenvironment by mediating immunosuppression. While exosomal miR-125-5p-derived melanoma enhances immunity by increasing the level of M1 phenotype markers IL-1β, CCL1, CCL2, and CD80 via targeting LIPA. *( +): increase, (−): decrease

Dendritic cells (DCs) represent the most potent professional antigen-presenting cells within the human body. They play a crucial role in initiating T-cell activation and orchestrating the central immune response. The regulation of cross-presentation in dendritic cells, alongside intercellular communication mediated by tumor-derived and endogenous exosomal miRNAs, can impact the maturation of DCs and their interactions with other immune cells [35, 138]. From these exosomal miRNAs, miR-212-3p from pancreatic cancer specifically targets the MHC II TF RFXAP, resulting in a diminished expression of HLA-DR, -DP, and -DQ molecules [139]. This interference negatively impacts the function of DCs (dendritic cells). In addition, miR-203 downregulates TLR4 (Toll-like receptor 4) and downstream cytokines in DCs affecting the activation of natural killer cells [140]. The miRNAs released from regulatory T cells (Tregs) possess the ability to influence the immune response. Specifically, the transfer of exosomal miR-150-5p and miR-142-3p to DCs induces a state of cellular refractoriness. This phenomenon of internalizing leads to an upregulation of IL-10 expression and a concurrent downregulation of IL-6 expression [141]. Moreover, exosomal miR-let-7d is transferred to T helper 1 (Th1) cells, resulting in the inhibition of Th1 cell proliferation and the suppression of IFN secretion. Notably, IFN, which is secreted by Th1 cells, serves as a pivotal player in the context of anti-tumor immunity [65]. In the context of breast cancer, miR-let-7i promotes DC maturation through the reduction of TGF-β, SOCS1, and KLRK1 levels, and the increase of IL-6, IL-7, IFNγ, and IL-1b [142]. Glioblastoma secretes miR-10a, and miR-21 affects the activation and differentiation of MDSCs (myeloid-derived suppressor cells) by targeting RORA and PTEN, respectively [143]. Exosomal miR-155 derived from chronic lymphatic leukemia (CLL) induce the reprogramming of monocytes to MDSCs resulting in immune suppression [144], the immunomodulatory mechanism shown in (Fig. 6) and (Table 2).

*Tumor progress is activated by extracellular matrix remodeling:* When malignant cells interact dynamically with their surrounding stroma leading to progress and growth of the tumor, this process is complicated. Fibroblasts represent the main source of connective tissues, ECM, and major cell types in the stromal [145]. The initiation and metastasis of cancer are suppressed by several inhibitory functions released by normal fibroblasts through direct communication between cells, paracrine signaling, and ECM integrity. Tumor-derived exosomal miRNAs have the capacity to initiate a series of tumor-promoting signals, triggering the transformation of normal fibroblasts (NFs) into cancer-associated fibroblasts (CAFs). This conversion stimulates the formation of new blood vessels, disrupts the homeostatic state of the extracellular matrix (ECM), and creates an optimal environment conducive to the proliferation and dissemination of cancer cells. These orchestrated effects contribute to the extensive progression of cancer within the affected tissues, shaping a landscape ideal for malignant growth [146]. The exosomal miRNAs play an important role in remodeling the ECM by changing the function of chondrocytes, osteoblasts, and certain epithelial cells. Therefore, this process affects angiogenesis, inflammatory response, and metabolic reprogramming.

**Fig. 7:**
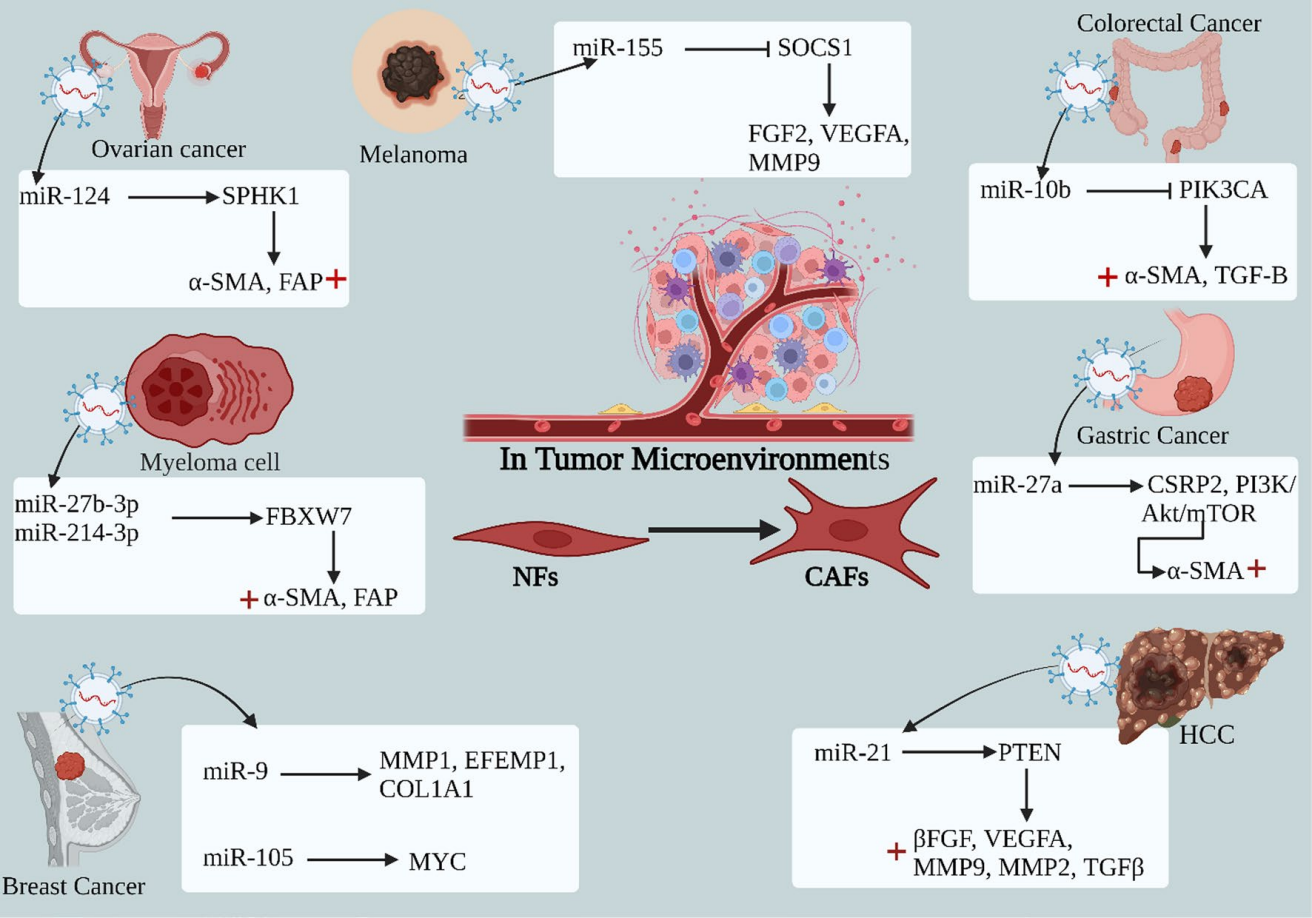
Remodeling of ECM mechanism by exosomal miRNA. The primary tumor cells release exosomes which were taken up by the normal fibroblasts (NFs) receptors, wherein the exosomal miR-124 derived ovarian cancer, miR-27B-3P, miR-214-3P derived myeloma, and miR-27a derived gastric cancer target the proteins (SPHK1, FBXW7, CSRP2), while miR-10b derived colorectal cancer inhibits PIK3CA leading to activation of fibroblast to cancer-associated fibroblasts (CAFs) by increasing the expression of α-SMA, and FAP and regulates CAFs migration and invasion. miR-155 derived melanoma inhibits SOCS1and promote activation of FGF2, VEGFA, MMP9 in CAFs. miR-21 derived HCC decrease PTEN leading to activation of PDK1/AKT signaling pathway and increasing expression of VEGF, MMP2, MMP9, βFGF and TGF-β in CAFs, promoting angiogenesis. Exosomal miRNA-derived breast cancer such as miR-9 activates NFs to CAFs by increasing MMP1, EFEMP1, COL1A1, and miR-105 inducing metabolic reprogramming of CAFs by activating MYC signal

The exosomes released by primary tumor cells were internalized by normal fibroblasts (NFs) through specific receptors. Within these exosomes, miR-124 derived from ovarian cancer, miR-27B-3P and miR-214-3P derived from myeloma, and miR-27a derived from gastric cancer target specific proteins, namely SPHK1, FBXW7, and CSRP2 [147–149]. Exosomal miR-10b derived from colorectal cancer inhibits PIK3CA, resulting in the activation of fibroblasts and their transformation into cancer-associated fibroblasts (CAFs) [150]. This transformation is characterized by an increase in the expression of α-SMA and FAP and plays a regulatory role in the migration and invasion of CAFs. In melanoma, miR-155 derived from the tumor inhibits SOCS1 and promotes the activation of the JAK2/STAT3 signaling pathway, increasing FGF2, VEGFA, and MMP9 levels in CAFs [151]. Similarly, in hepatocellular carcinoma (HCC), miR-21 derived from the tumor reduces PTEN levels, leading to the activation of the PDK1/AKT signaling pathway and an increase in the expression of VEGF, MMP2, MMP9, βFGF, and TGF-β in CAFs, thereby promoting angiogenesis [152, 153]. Additionally, exosomal miRNAs derived from breast cancer, such as miR-9, activate NFs to transform into CAFs by upregulating MMP1, EFEMP1, and COL1A1 [154]. Moreover, miR-105 induces metabolic reprogramming of CAFs by activating the MYC signaling pathway [155]. The mechanisms of ECM remodeling by exosomal miRNAs are summarized in (Fig. 7) and (Table 2).

## Conclusion

More than four decades, the groundbreaking discovery of exosomes within immature red blood cells by Stahl and Johnstone marked a pivotal milestone [17, 156, 157]. These small vesicles, known as exosomes, encapsulate a diverse cargo that reflects their cell of origin. Fascinatingly, exosomes possess the remarkable capability to transfer their contents between cells, endowing them with significant roles in both physiological and pathological conditions. Of particular interest within the exosomal cargo are microRNAs (miRNAs), which serve as key messengers in signal transduction between immune cells, including dendritic cells, macrophages, lymphocytes, neutrophils, mast cells, and non-immune cells. These miRNA-loaded exosomes orchestrate vital processes such as the control of dendritic cell development, maturation, and function, as well as the polarization of macrophages. In the context of colitis, exosomal miRNAs derived from regulatory T cells effectively suppress Th1 cells, illustrating the profound impact of miRNAs as crucial modulators of the immune system. Furthermore, exosomes emerge as the principal mediators responsible for the heterogeneous nature of the tumor microenvironment (TME). Through the transfer of their contents between tumor cells and the TME, exosomes facilitate critical adaptations of tumor cells to the low oxygen levels of the TME, promoting angiogenesis or facilitating metastasis to more favorable environments. Remarkably, exosomal miRNAs secreted by tumor cells actively reshape the extracellular matrix and potentiate tumor cell spread by activating cancer-associated fibroblasts. Consequently, exosomal miRNAs exert an immunomodulatory influence within the dynamic landscape of the TME. In conclusion, the captivating realm of exosomes and their cargo, particularly miRNAs, holds immense potential for understanding and manipulating intricate cellular interactions. These findings enhance our comprehension of fundamental biological processes and open up exciting avenues for therapeutic interventions in various pathological contexts.

## Data Availability

Not applicable.

## Abbreviations

EVs: Extracellular vesicles
MVBs: Multivesicular bodies
ILVs: Intraluminal vesicles
ESEs: Early-sorting endosomes
LSEs: Late-sorting endosomes
UTR: Untranslated region
ORF: Open reading frame
HDL: High-density lipoprotein
pri-miRNA: Primary miRNA
nSMase2: Neural sphingomyelinase 2-dependent
hnRNP: Heterogeneous nuclear ribonucleoprotein
RISC: RNA-induced silencing complex
GW182: Glycine-tryptophan protein of 182 kDa
DCs: Dendritic cells
BM: Bone marrow
PAMPs: Pathogen-associated molecular patterns
LPS: Lipopolysaccharide
BMDCs: Bone marrow-derived DCs
snoRNAs: Small nucleolar RNAs
MSC: Mesenchymal stem cell
Mregs: Regulatory macrophages
DSS: Dextran sulfate sodium
Treg: T regulatory
TNF-α: Tumor necrosis factor-alpha
NF-κB: Nuclear factor kappa B
TXNIP: Thioredoxin-interacting protein
TAMs: Tumor-associated macrophages
PTEN gene: Phosphatase and tensin homolog gene
SP: Suppressor phenotype
PC: Pancreatic cancer
ER: Endoplasmic reticulum
SOX-4 miRNA: SRY-box transcription factor 4 mRNA
IS: Immune synapsis
Th1: T helper 1
APCs: Antigen presenting cells
BCRs: B cell receptors
BAFF: B-cell activating factor
TLR7: Toll-like receptor 7 polymorphism
OCLN: Occludin
CLDN8: Claudin 8
CNS: Central nervous system
HCC: Hepatocellular carcinoma
ROS: Oxygen species
NO: Nitric oxide
BSMC: Bronchial smooth muscle cells
SAEC: Small airway epithelial cells
hUCMSC: Human umbilical cord mesenchymal stem cell
IL: Interleukin
NKs: Natural killer cells
PKCα: Protein kinase C alpha
NETosis: Neutrophil extracellular traps
dSSc: Diffuse cutaneous systemic sclerosis
BMECs: Brain microvascular endothelial cells
PMNs: Polymorphonuclear neutrophils
ALI: Acute lung injury
ARDS: Acute respiratory distress syndrome
TME: Tumor microenvironment
MMPs: Matrix metalloproteinases
HIF-1α: Hypoxia inducible factor-1α
VEGF: Vascular endothelial growth factor
NFs: Normal fibroblasts
NSCLC: Non-small cell lung cancer
MDSCs: Myeloid-derived suppressor cells
CLL: Chronic lymphatic leukaemia
CAFs: Cancer associated fibroblasts
SPHK1: Sphingosine kinase 1
TAMs: Tumor associated macrophages
HLSCs: Human liver stem-like cells
HUVECs: Human umbilical vein endothelial cells
PHD1 and PHD2: Prolyl hydroxylase 1 and 2
NKs: Natural killer cells
EOC: Epithelial ovarian cancer
MDSCs: Myeloid-derived suppressor cells
α-SMA: α-Smooth muscle actin
FAP: Fibroblast activating protein
